# Fellowships, Grants, & Awards

**Published:** 2004-11

## Obesity and the Built Environment

In the United States, obesity and overweight have risen to an epidemic rate during the past 20 years and are among the most important health challenges of our time. Obesity in adults is associated with increased risk of a number of diseases and metabolic abnormalities; most of the conditions associated with obesity are also associated with increasing age. Overweight among children aged 6–19 years currently stands at 15%. Risk factors for heart disease, such as high cholesterol and high blood pressure, occur with increased frequency in overweight children compared to children with a healthy weight; type 2 diabetes mellitus, previously considered an adult disease, is increasingly observed in overweight children. Obesity coupled with impaired mobility can easily lead to an increase in functional limitations and a decrease in the quality of life.

This request for applications (RFA) will support projects that delineate the significance and impact of the built environment on overweight and obesity by enhancing our understanding of the roles played by city and regional planning, housing, transportation, media, access to healthy foods, and availability of public and green spaces (e.g., playgrounds, walking paths) as determinants of physical activity and nutritious dietary practices. This initiative will support studies in two specific areas related to the built environment and obesity: 1) understanding the role of the built environment in causing/exacerbating obesity and related comorbidities, and 2) developing, implementing, and evaluating prevention/intervention strategies that influence parameters of the built environment in order to reduce the prevalence of overweight, obesity, and comorbidities.

The built environment encompasses all buildings, spaces, and products that are created or modified by people. It includes homes, schools, workplaces, parks/recreation areas, greenways, business areas, and transportation systems. It extends overhead in the form of electric transmission lines, underground in the form of waste disposal sites and subway trains, and across the country in the form of highways. It includes land-use planning and policies that impact our communities in urban, rural, and suburban areas.

Currently, there is insufficient research that delineates the influence of the built environment on nutritional factors and physical activity. One intended outcome of this program is the development of models of health-promoting communities that provide access to a wide variety of healthful foods and physical activity patterns, and how this impacts overweight and obesity.

These community-based environmental interventions would serve as models for management and prevention of overweight, obesity, and comorbidities across other communities in need of change. Of particular interest are studies conducted in vulnerable populations (such as children, the aging population, low-socioeconomic-status [SES] communities, racial/ethnic minorities, and persons with disabilities that require use of assistive mobility devices such as wheelchairs and prostheses).

Many educational programs targeted to individuals to effect changes in obesity and weight gain have not been successful. However, environmental changes that reinforce factors supporting healthy lifestyles and reduce barriers to healthy lifestyles may serve to diminish health disparities. Access to healthy foods and opportunities to impact physical activity are factors that have been considered at both the community and individual levels. Approaches that modify the environment to promote healthful eating practices, increase physical activity, and decrease sedentary behaviors offer the potential for safe and effective programs for obesity prevention that could be widely disseminated.

Because of the wide range of issues to be addressed and the diversity of communities, this RFA requires interdisciplinary partnerships. These teams must consist, at a minimum, of a scientist with expertise in health research, a clinical specialist, and an expert on planning, design, or transportation. Other scientists and experts as well as those with scientific expertise in diseases and conditions that are comorbidities of obesity should be considered to complement, but will not substitute for, those mentioned above. Partnerships with state and local health departments working in the area of obesity prevention and control through promotion of improved physical activity and nutrition may also be considered.

Investigators applying to this RFA may propose collaborations with organizations/institutions such as schools; supermarkets; restaurants; religious organizations; recreation facilities; industry, governmental, public health, or community groups; worksites; and so forth, to develop approaches that could potentially be translated into larger-scale prevention/intervention programs.

Research on the connections between the built environment and health has shown that the burden of illness has been greater on lower-SES strata and minority/vulnerable populations, including persons who have impaired mobility. Increased environmental pollutants have resulted in lifestyle changes, especially with regard to diet and physical activity. For example, the increased pollution in Alaskan waters has led to some Native American communities substituting their traditional diets with more foods bought at the grocery stores. This also has had a negative impact on their traditional fishing and hunting activities. Lower-SES communities usually have limited access to quality housing and live in neighborhoods that do not facilitate outdoor activities and provide an uneven mix of healthy and unhealthy food outlet choices such as few supermarkets but many low-cost restaurants. Inequities in construction and maintenance of low-income housing——especially for racial/ethnic minorities, older persons, and immigrants—have resulted in insufficient housing, poor-quality housing, overcrowding, and higher levels of population density and health problems. Also, studies have consistently shown an association between a deteriorated physical environment and higher rates of crime, making neighborhoods less safe for walking and in some cases resulting in greater social isolation.

Most studies to date have solely focused their efforts in the urban environment, while there are several unattended issues in rural areas. For example, greater pesticide use in rural areas has resulted in people remaining indoors for longer periods of time and decreasing non-motorized travel. One of the consequences of motorized ways of living in rural areas has been decreased levels of physical activity and concomitant increases in obesity.

Though the built environment is one of our most important habitats, current research in the area has focused mainly on the challenges of balanced transportation, urban sprawl, air pollution due to increased traffic, and the diminishing natural environment. However, more concerted research is needed to identify mechanisms by which the built environment adversely and positively impacts health, and to develop appropriate interventions to reduce or eliminate harmful health effects.

Some recent research explores the effect of improved built environments or sustainable communities on physical activity, asthma, obesity, cardiovascular disease, lung cancer mortality, and mental health. The President’s Council on Sustainable Development in 1993 (Executive Order 12852) offered a working definition for sustainable communities as “healthy communities where natural and historic resources are preserved, jobs are available, sprawl is contained, neighborhoods are secure, education is life-long, transportation and health care are accessible, and all citizens have opportunities to improve the quality of their lives.” While much research indicates the negative health impact of a poor built environment, there is very limited research on the health benefits of promoting sustainable communities to reduce overweight, obesity, and related comorbidities. The sparse research on sustainable communities suggests that diligent planning is needed to create an environment that is conducive to the mental and physical well-being of humans as well as the natural environment.

This RFA will support both R21 and R01 mechanisms. To understand the wide variety of issues relating to the built environment and obesity, studies that combine qualitative and quantitative methodologies are strongly encouraged. Studies can be conducted at the micro (individual/familial), meso (interpersonal and community), and macro (societal, policy) levels. Intervention projects must incorporate multilevel approaches. The Community Guide developed by the Community Preventive Services Task Force (appointed by the director of the Centers for Disease Control and Prevention) provides recommendations regarding population-based interventions for physical activity (see http://www.thecommunityguide.org/pa/default.htm). Intervention projects supported by this RFA must build upon and advance these recommendations and should not duplicate the Task Force efforts. Studies should develop and include new and improved objective measures that link the built environment to biology wherever appropriate, such as serum glucose, endothelium-dependent dilation, cholesterol, insulin, energy balance, oxidative stress, body composition, and lipid profile.

The Surgeon General’s Report on overweight and obesity (http://www.surgeongeneral.gov/topics/obesity/calltoaction/CalltoAction.pdf.) indicates that environmental modifications offer the best opportunity for the treatment and prevention of obesity. Given this, multisite and multilevel environmental interventions including schools, worksites, communities, and a variety of larger social settings would be appropriate. Single site–based interventions have been shown to be effective in improving diet and physical activity levels of children for a short period of time through changes in school lunch and vending machine contents and levels of physical activity. Thus, the most effective strategy would be studies that address both energy intake and expenditure and combine different levels and settings. Across all settings, interventions submitted in response to this RFA may address food access and availability, opportunities for physical activity, and the policies that connect energy intake and expenditure to the built environment.

Evaluation is an essential component for all intervention studies, and proposed studies should include both process and outcome measures. The latter could also include appropriate biological measures. While it is important that both quantitative and qualitative measures are included, wherever possible the adoption of objective measures is encouraged.

The R21 mechanism (see http://grants.nih.gov/grants/guide/pa-files/PA-03-107.html) is limited to two years of funding and is intended to encourage new exploratory/developmental research projects by providing support for the early stages of their development. For example, such pilot/exploratory projects could assess the feasibility of a novel area of investigation or a new experimental system that has the potential to enhance health-related research. Applications for R21 awards should describe projects distinct from those supported through the traditional R01 mechanism. For example, long-term projects or projects designed to increase knowledge in a well-established area will not be considered for R21 awards. Applications submitted under the R21 mechanism should be exploratory and novel and may include development of new methods and measurements. Under these provisions, these R21 proposals should evaluate the validity and reliability of the measures and when appropriate also ensure that they are linguistically and culturally relevant.

Some areas of interest for R21 submissions include but are not limited to the following: 1) at the macro level, the development and validation of reliable measures/indicators for assessing the means by which the built environment impacts food availability/access and physical activity to promote healthy behaviors and lifestyles and reduce obesity and related comorbidities; 2) at the meso level, the development of measures of neighborhood characteristics that promote healthy lifestyles and mediate the effect of the built environment on overweight, obesity, and related comorbidities; 3) at the micro level, the development of valid and reliable measures for behaviors that moderate obesity such as physical activity, nutrient intake, etc.; it is also important that these measures be linguistically and culturally validated; 4) feasibility studies to identify and quantify risk factors and variables related to the built environment that mediate and moderate built environment health effects for public health interventions and outcomes to reduce overweight, obesity, and related comorbidities; 5) pilot studies that assess the impact of state, local, and institutional policy changes in the built environment on overweight, obesity, and related comorbidities (these could be an evaluation of natural experiments such as comparing differently designed communities, or examining before-and-after events, such as physical activity and driving levels before and after a new transit system is opened and nutritional changes with the removal of unhealthy foods from schools); 6) studies to identify environmental and social conditions of people’s work and living choices that may influence physical activity and dietary intake; for example, whether healthy people choose to live in healthy environments or healthy environments differentially attract healthy people; 7) evaluate the health impact assessment instrument as an appropriate and useful tool for local and state health officials to provide information to decision makers about the health consequences of proposed built environment projects and policies; or 8) studies, such as the above examples, that include or focus on obesity concerns and needs of persons who have impaired mobility and use assistive devices such as wheelchairs and lower-limb prostheses.

This RFA uses just-in-time concepts. It also uses the modular budgeting as well as the nonmodular budgeting formats (see http://grants.nih.gov/grants/funding/modular/modular.htm). Specifically, if you are submitting an application with direct costs in each year of $250,000 or less, use the modular budget format. Otherwise, follow the instructions for nonmodular budget research grant applications. If you are submitting an R21 application, use the modular budget format. This program does not require cost sharing as defined in the current NIH Grants Policy Statement at http://grants.nih.gov/grants/policy/nihgps_2003/NIHGPS_Part2.htm.

The participating institutes intend to commit approximately $5 million in fiscal year 2005 to fund 10–12 new grants in response to this RFA. R01 applications may request a project period of up to five years and a budget for direct costs of up to $400,000 per year, excluding fiscal and administrative costs (http://grants.nih.gov/grants/guide/notice-files/NOT-OD-04-040.html). R21 applications may request a project period of up to two years with a combined budget for direct costs of up to $275,000 for the two-year period. For example, requests can be $100,000 in the first year and $175,000 in the second year.

Letters of intent must be received by 17 November 2004. Applications must be received by 17 December 2004. The complete version of this RFA is available online at http://grants.nih.gov/grants/guide/rfa-files/RFA-ES-04-003.html.

Contact: Shobha Srinivasan, NIEHS, PO Box 12233, MD EC-21, 111 T.W. Alexander Dr, Research Triangle Park, NC 27709 USA, 919-541-2506, fax: 919-316-4606, e-mail: sriniva2@niehs.nih.gov; Deborah H. Olster, Office of Behavioral and Social Sciences Research, NIH, Bldg 1, Rm 256, One Center Dr, Bethesda, MD 20892-0183 USA, 301-451-4286, fax: 301-402-1150, e-mail: olsterd@od.nih.gov; Harold W. Kohl, III, CDC, National Center for Chronic Disease Prevention and Health Promotion, 4770 Buford Hwy NE, MS K-46, Atlanta, GA 30341-3717 USA, 770-488-5481, fax: 770-488-5473, e-mail: hkohl@cdc.gov; Andrew Dannenberg, CDC, National Center for Environmental Health, 4770 Buford Hwy NE, MS F-30, Atlanta, GA 30341 USA, 770-488-7108, e-mail: acd7@cdc.gov; Gilman D. Grave, NICHD, 6100 Executive Blvd, Ste 4B-11, Bethesda, MD 20892-7510 USA, 301-496-5593, fax: 301-480-9791, e-mail: gg37v@nih.gov; Louise C. Masse, NCI, 6130 Executive Blvd, MSC 7335, Executive Plaza North, Rm 4076, Bethesda, MD 20892-7335 USA, 301-435-3961, fax: 301-480-2087, e-mail: massel@mail.nih.gov. Reference: RFA No. RFA-ES-04-003

## Novel Approaches to Enhance Animal Stem Cell Research

The purpose of this program announcement (PA) is to encourage research to enhance animal stem cells as model biological systems. Innovative approaches to isolate, characterize, and identify totipotent and multipotent stem cells from nonhuman biomedical research animal models, as well as to generate reagents and techniques to characterize and separate those stem cells from other cell types, is encouraged. Embryonic and other stem cells are valuable biomedical research models for the study of biological and disease processes and the creation of disease models. In addition, these cells hold promise as model systems for development of therapeutics and of replacement tissues.

Thus far, embryonic stem cells have been isolated from some biomedically important nonhuman research models. In addition, stem cells with a more restricted potential have been characterized from post-embryonic tissue types. However, research is needed to provide for a full array of totipotent and multipotent stem cells from nonhuman biomedical research animal models, as well as to provide the research tools to identify, characterize, and purify those cells.

This PA supports the isolation and characterization of embryonic and other multipotent stem cells in a variety of nonhuman animal species. Examples of research areas appropriate to this PA include, but are not limited to, projects to 1) expand the number of nonhuman animal model systems in which embryonic stem cells are available; 2) identify, isolate, culture, and characterize multipotent stem cell populations derived from nonhuman embryonic stem cells; 3) identify, isolate, culture, and characterize multipotent stem cells from postfetal tissue types; 4) generate and use panels of markers for stem cell attributes common across species for use in characterization and isolation of stem cells in a range of animal species or tissues; and 5) create universal methods of culture to maintain the undifferentiated state of embryonic or other characterized multipotential stem cells across nonhuman animal species.

Projects supported by the National Center for Research Resources under this PA are intended to generate research tools, reagents, or stem cells of utility to research on a broad range of tissue or cell types and of interest to more than one categorical or disease-oriented NIH institute or center. Projects that will focus on research on tissues or disease processes specific to the mission of an institute or center should be directed to the respective facility.

This PA will use the NIH R01 and R21 award mechanisms. As an applicant, you will be solely responsible for planning, directing, and executing the proposed project. R21 applications should meet the requirements for this mechanism as recently redefined in PA-03-107, available at http://grants.nih.gov/grants/guide/pa-files/PA-03-107.html. In brief, by using the R21 mechanism, the NIH seeks to foster the introduction of novel scientific ideas, model systems, tools, agents, targets, and technologies that have the potential to substantially advance biomedical research. These studies may involve considerable risk but may lead to breakthroughs, developments, or applications that could have a major impact on a field of biomedical, behavioral, or clinical research.

Applications for R21 awards should describe projects distinct from those supported through the traditional R01 mechanism. For example, long-term projects or projects designed to increase knowledge in a well-established area will not be considered for R21 awards. Applications submitted under this mechanism should be exploratory and novel. These studies should break new ground or extend previous discoveries toward new directions or applications.

Applications for R21 awards may request a project period of up to two years with a combined budget for direct costs of up to $275,000 for the two-year period. The request should be tailored to the needs of the project. Normally, no more than $200,000 may be requested in any single year.

This PA uses just-in-time concepts. It also uses the modular budgeting as well as the nonmodular budgeting formats (see http://grants.nih.gov/grants/funding/modular/modular.htm). Specifically, if you are submitting an application with direct costs in each year of $250,000 or less, use the modular budgeting format. Otherwise, follow the instructions for nonmodular budgeting research grant applications. This program does not require cost sharing as defined in the current NIH Grants Policy Statement at http://grants.nih.gov/grants/policy/nihgps_2003/NIHGPS_Part2.htm.

Applications must be prepared using the PHS 398 research grant application instructions and forms (rev. 5/2001). Applications must have a Dun and Bradstreet Data Universal Numbering System (DUNS) number as the Universal Identifier when applying for federal grants or cooperative agreements. The DUNS number can be obtained by calling 1-866-705-5711 or through the website at http://www.dunandbradstreet.com/. The DUNS number should be entered on line 11 of the face page of the PHS 398 form. The PHS 398 document is available at http://grants.nih.gov/grants/funding/phs398/phs398.html in an interactive format. For further assistance, contact GrantsInfo by calling 301-435-0714 or e-mailing GrantsInfo@nih.gov.

Applications submitted in response to this PA will be accepted at the standard application deadlines, which are available at http://grants.nih.gov/grants/dates.htm. Application deadlines are also indicated in the PHS 398 application kit.

Contact: For the complete listing of contacts, please consult the full PA, available online at http://grants1.nih.gov/grants/guide/pa-files/PA-04-125.html. Reference: PA No. PA-04-126

